# Getting the Problem Definition Right: The Radical Right, Populism, Nativism and Public Health Comment on "A Scoping Review of Populist Radical Right Parties’ Influence on Welfare Policy and its Implications for Population Health in Europe"

**DOI:** 10.34172/ijhpm.2020.143

**Published:** 2020-08-01

**Authors:** Benjamin De Cleen, Ewen Speed

**Affiliations:** ^1^Vrije Universiteit Brussel (VUB), Brussels, Belgium.; ^2^School of Health and Social Care, University of Essex, Colchester, UK.

**Keywords:** Radical Right, Populism, Nativism, Health Policy, Europe

## Abstract

Building on Rinaldi and Bekker’s scoping review of articles on the impact of populist radical right (PRR) politics on welfare and population health, this short article formulates three pointers towards a framework that might help structure future research into PRR, populist politics more generally, and coronavirus disease 2019 (COVID-19) and other health issues. First, we discuss the centrality of welfare chauvinism to the PRR’s impact on health, taking this as a cue for a broader reflection on the importance on distinguishing between the nativist and populist dimensions of PRR politics. Secondly, we turn our attention to the potential moderating effect of the PRR’s welfare chauvinism on the welfare cuts proposed by their right-wing coalition partners, comments we see as pointing to the need to focus on nativist, populist, neoliberal and other threats to welfare policy more generally, rather than on the PRR only. Thirdly, we reflect on the paradoxical nature of welfare chauvinism – its negative consequences for the health of the ‘own people’ it proclaims to defend – as a starting point for a brief discussion of the need to consider carefully the not-so-straightforward relation between the PRR’s political rhetoric, its (impact on) policy and institutions, and the outcomes of such policy.

 Never has the impact of populist radical right (PRR) politics on health policy been higher on the public agenda than since the outbreak of the coronavirus disease 2019 (COVID-19) pandemic. US President Trump’s and Brazilian President Jair Bolsonaro’s management of the crisis in particular has attracted much (negative) attention from media and commentators.

Written before the outbreak of the COVID-19 pandemic, Chiara Rinaldi and Marleen P.M. Bekker’s article^1^ presents a scoping review of 15 articles about the impact of populist radical parties on welfare policy and population health. A few years from now, such a scoping review will have to cover a far larger number of articles as wave of academic publications on COVID-19 and populism and the radical right is likely coming our way. In this short piece we comment on the Rinaldi and Bekker article. We would also like to take the opportunity to build on Rinaldi and Bekker’s work to formulate a few potential indicators towards a framework that might help structure future research into populism, the radical right and COVID-19 and other welfare and health issues.

 Rinaldi and Bekker’s review of existing work almost unavoidably produces rather scattered results. This is due to the limited number of articles on the PRR and welfare policy, the diversity of approaches in the articles they collected, the differences between national contexts, as well as the inherent difficulty of measuring the impact on welfare policy and its outcomes of a collection of PRR parties that show quite some diversity. Still, Rinaldi and Bekker’s article points to a number of significant findings.

 In our comment we reflect on three of Rinaldi and Bekker’s insights that we consider to be of major importance to further research into the PRR, welfare policy and population health. In a first section we discuss the centrality of welfare chauvinism to the PRR’s impact on health, taking this as a cue for a broader reflection on the importance on distinguishing between the nativist and populist dimensions of PRR politics. In a second section we turn our attention to Rinaldi and Bekker’s comments on the potential moderating effect of the PRR’s welfare chauvinism on the welfare cuts proposed by their right-wing coalition partners, comments we see as pointing to the need to focus on nativist, populist, neoliberal threats (as well as other threats to welfare more generally), rather than on the PRR only. In a third and final section we take Rinaldi and Bekker’s insightful remark about the paradoxical nature of welfare chauvinism – its negative consequences for the health of the ‘own people’ it proclaims to defend – as a starting point for a brief discussion of the need to consider carefully the not-so-straightforward relation between the PRR’s political rhetoric, its (impact on) policy and institutions, and the health outcomes of such policy.

## 1. Welfare Chauvinism and the Nativism and Populism of the Radical Right

 The main conclusion Rinaldi and Bekker draw from their review is that “[w]elfare chauvinism is the most prominent channel through which PRR parties could adversely affect population health and health equity in Europe, as welfare chauvinistic policies have the potential to directly affect access to welfare provisions for vulnerable (immigrant) groups” (p. 8). The impact of PRR parties in government, they find from their review, works through direct exclusion of migrants from welfare benefits (an example they give is Sweden reducing aid to vulnerable EU immigrants) and, more often, indirectly, “through policies that are targeted at the entire population but affect ‘undeserving’ (immigrant) populations disproportionally, such as policies that restrict eligibility to unemployment benefits” (p. 6). Moreover, even where PRR parties are not in government, Rinaldi and Bekker indicate, “mainstream parties of either side of the political spectrum were found to allow or even propose exclusionary welfare policies as a reaction to PRR electoral success” (p. 9).

As Rinaldi and Bekker rightly indicate (p. 6), welfare chauvinism is rooted in the PRR’s nativism (an exclusionary ethnic nationalism that favours the ‘native’ population) and – less directly so, we would say – from their authoritarian defence of “those ‘morally deserving’ of support” (p. 6). Without stating it so explicitly, Rinaldi and Bekker’s review confirms that PRR politics are first and foremost nativist (and authoritarian) rather than populist.^2^ This is a crucial point, and a point where Rinaldi and Bekker avoid some of the common problems in discussions on the PRR. All too often, PRR parties are labelled as simply ‘populist,’ especially in media coverage and political commentary.^3^

There are several problems with this equation between ‘populist radical right’ and ‘populist.’ First of all, it obscures the fact that the ideological core of PRR politics is nativism, and not populism.^4^ Secondly, the use of the term ‘populism’ *per se* tends to conflate radical right and radical left populisms, ignoring the ideological divide between such left and right types of populist politics – a cleavage that clearly shows in the difference between the exclusionary welfare chauvinism of the right and the ‘inclusionary populist’ approach to welfare of the left.^5^ And thirdly, labelling the PRR as simply ‘populist’ tends to conceptually conflate populism with exclusionary ethnic nationalism (or nativism), seeing the exclusion of national ‘others’ as intrinsic to populism. This misses the crucial analytical distinction between populism, nativism and nationalism more broadly.

We see populism not as a thin ideology (as Rinaldi and Bekker do, following Cas Mudde) but as a political logic that revolves around the bringing together of different demands and groups through the discursive construction of a vertical distinction between ‘the people’ as a large powerless group, or underdog, and ‘the elite’ as a small group whose power is illegitimate because they do not represent ‘the people,’ with populist political forces claiming to represent ‘the people’ against that illegitimate elite. Nationalist politics, by comparison, revolve around the claim to represent the people-as-nation, defined through a differentiation based on territory, language, shared history, and ethnicity that is horizontal, in/out (which can of course have elements of superiority/inferiority).^6-8^ This definition of nationalism allows for more or less inclusive/open or exclusive/closed definitions of the nation and the more or less radical conclusions draws from that in terms of both migration policy (expelling migrants, integration, cultural diversity) and national independence (from an independent state to cultural autonomy within a plurinational state).^6^ Nativism is a term used for a particular breed of nationalist politics that revolve around an ethnic and exclusionary definition of the nation and the defence of the ‘natives’ against national outsiders; even if the distinction between ethnic-nativist and more open and inclusionary, ‘civic’ nationalist politics is by no means clear-cut. In any case, a clear analytical distinction between nationalism (of whatever kind) and populism is needed in order to be able to grasp the intricate entanglement of populism and nationalism in PRR politics (or in other kinds of politics, for that matter).

The populist logic per se is never solely politically of the right, the left or the centre. The ways in which populism plays *out* is contingent upon the actors using that logic and the context in which this occurs. It is the underpinning social rationality which the populist tropes are mobilised in support of that might more readily be characterised as being of the right, the left or the centre. In this, we take a direct line from Laclau’s work on the populist discursive political logic when he argues that “populism’s relative ideological simplicity and emptiness should be approached in terms of what those processes of simplification [of the political space into ‘people’ versus ‘elite’] and emptying attempt to perform, that is to say, the social rationality they express.”^9^ This means that the notion of populism is never sufficient to characterize the politics of any political actor and that analyses should always consider what kind of political a populist political logic is used to promote, and how the populist categories ‘the people’ and ‘the elite’ acquire meaning in relation to other ideological categories such as ‘the nation’ or ‘social class.’

As such, the near absence of reflections about populism in Rinaldi and Bekker’s work – whilst avoiding common pitfalls in speaking about populism – is also a limitation. There are two primary reasons for this. One is that the PRR’s populist claim to represent the people-as-underdog and oppose ‘the elite’- whilst not ideologically central – is a crucial element of its rhetoric, and a major factor in its electoral appeal. PRR rhetoric and success hinge on the entanglement between defending national sovereignty, excluding migrants and foreigners, and claiming to represent the ordinary people against the elite. This populism is also one of the main strategies for legitimizing nativist positions as representing the ‘voice of the people,’ with that claim being accepted across much of the political spectrum and thus exerting influence on the positions of the PRR’s contenders.^10^

Secondly, whilst we should be very careful not to equate populism with ‘post-truth politics’ or anti-expertise positions,^11,12^ it is clear that the radical right has regularly used populist strategies to delegitimize the expertise of journalists, academics and others, also in the context of health. An example here would be the number of PRR parties allied with anti-vaccination movements both at national and international levels.^5^

There is no necessary correspondence between populism and negative impacts on health. Left populisms have often included demands for welfare provisions and accessible healthcare.^13^ It is even possible that in some instances the populism of the radical right might have positive effects on welfare policies (for the natives) as well. Indeed, a third reason why populism needs to be included in reflections on the PRR and health is that the PRR’s populist claim to represent the ‘ordinary people,’ in close combination with its welfare chauvinism might sometimes urge them to take pro-welfare stances (albeit reserved for the ‘native’ population). The Belgian Vlaams Belang is a clear example here (at least in its rhetoric, see point 3 below), the party consistently offering voters the choice between more welfare for ‘the own people’ and financing migration and asylum and solidarity with the poorer South of the country. Moreover, Rinaldi and Bekker write that

 “[m]ost PRR parties in Europe have entered the executive government office in centre-right government coalitions with ambitions for welfare retrenchment. PRR parties thus face a trade-off between supporting the retrenchment proposals of their coalition partners to establish a coalition agreement (office-seeking behaviour) or enforcing welfare policies that benefit their electorate more directly (vote-seeking behaviour)” (p. 7).

 This passage is remarkable in that it suggests that the PRR is not only a threat to welfare and health through its exclusion of ‘non-natives,’ its welfare chauvinism and populism in some cases also constitute a counter-weight to the welfare cuts proposed by their right-wing contenders and coalition partners. Whilst Rinaldi and Bekker’s article is focused on the PRR, their argument here can be said to constitute a warning against an exclusive focus on the PRR as a threat to healthcare.

## 2. Against an Exclusive Focus on the Populist Radical Right

There is no doubt that the PRR’s impact on population health merits dedicated attention. But this should not be to the detriment of critical analysis of the record of parties in the political center, whose often neoliberally inspired welfare cuts have likely impacted population health negatively much more so than the PRR has so managed to do (so far). Moreover, even if our focus is on the impact of nativism and populism (rather than neoliberalism), we should be careful not to fix our analysis too much at the point of the PRR as parties. If what we are interested in is ideology, policy and actual impact on health, then the performance of nativism and populism, by and through various political actors merits closer analysis, rather than the impact of a certain set of parties *per se*.

 Why do we characterise this as a risk? By conceiving of nativism and populism as a property of the PRR, in part this functions to make such politics marginal, and to categorise them as outside of the political mainstream. The risk is that this then fails to hold to account many of those mainstream politicians, who draw from those self-same nativist and populist tropes. Only when they do so, it is not regarded as populist or dangerously nativist, because it does not occur in a marginal political context.

For example, consider the role of nativism and populism within the UK Conservative Party in the Brexit campaign. This party is not a PRR organisation, yet it consistently utilised nativist and populist tropes in the Brexit referendum. In the context of health, they blamed so-called ‘health tourists’ for the state of the beleaguered UK National Health Service when the evidence suggests that the United Kingdom is actually a net exporter of health tourists to the EU.^14^ This trope functions to conflate questions of access to healthcare with questions of immigration, constructing and invoking a ‘threat’ that immigration poses for the native population in accessing healthcare. This example also leads onto our final argument.

## 3. Health Policy Context vs. Political Context

 A third and final point we would like to make hinges on the distinction between policy and politics and on the complexity of the relation between politics, policy and outcomes. Rinaldi and Bekker write about the negative health outcomes of welfare chauvinism that:

 “Both universal access to healthcare and other redistributive welfare provisions, such as pensions and unemployment benefits, have been associated with increased population health either directly or indirectly. This confirms the idea that PRR parties could pose a threat to population health due to their exclusionary policy agenda, especially since positive effects of welfare chauvinism for the native population are not clear” (p. 8).

 They conclude that “[w]elfare chauvinism might thus represent a paradox in which it harms its very own proponents, especially the most vulnerable (eg, people who are unemployed)” (p. 8). This is a very significant point: not only does welfare chauvinism negatively impact the nativist outsiders, it might also harm the ‘own people’ it is supposed to benefit.

 Consider the context of tuberculosis in the United kingdom in the past eight years. In that timeframe we see a centre right government actively operating a ‘hostile environment’ principle, designed to create a hostile environment to each and every migrant (regardless of their legal status). This principle plays out in the context of healthcare primarily in terms of questions of access. The social rationality is one where a beleaguered health service is seen to be struggling to provide healthcare to those entitled to it, because too many people (who are not entitled to free, at the point of use healthcare) are using it, either as legal migrants, illegal migrants or health tourists. This functions to create ‘legitimate’ welfare chauvinist limitations on access to what is ostensibly a universal healthcare service – in theory making the healthcare system more accessible to those who are ‘entitled’ to access it.

But this welfare chauvinism has other public health consequences. In parallels to the current UK COVID-19 lockdown, the hostile environment policies (constructed with a rationality predicated on protecting healthcare for all those entitled to it), simultaneously work to create a very real public risk. Many immigrant groups in London experienced a spike in rates of tuberculosis following the hostile environment policies.^15^ This is attributed to these groups being unable to seek competent healthcare, for fear of arrest and deportation. In the context of what is a highly contagious disease, government policy can be seen to be actively working against the public health interest, and in fact, could be argued to be putting more strain on the beleaguered health service (through increased levels of mortality and morbidity) which it purports to want to protect.

 As such there is a fundamental contradiction between welfare chauvinism and actual health policy outcomes. These welfare chauvinist policies once the limits of this self-same chauvinism are considered next to the ‘hard’ consequences of that logic, including for the ‘natives,’ eg, increased mortality and morbidity at a population level. In this context, it becomes difficult to argue the sanctity of welfare chauvinism as an organising principle for health policy. In fact welfare chauvinism can be shown to work against the implementation of effective health policy. Instead of taking welfare chauvinism at face value, there is a need for analyses to demonstrate the ways in which welfare chauvinism is performed in very simplified and rhetorically empty ways, ways which work to prevent it being held to account against the cold hard facts that more people have become ill or died because of these policies.

## Conclusion

 Rinaldi and Bekker’s scoping review shows that much work remains to be done if we are to arrive at a fuller understanding of the impact of PRR politics on welfare policy and health, and Rinaldi and Bekker already indicate some significant areas of further research. With the COVID-19-pandemic, there is little doubt that much work will be done on this in the next few years. In this article, we have suggested three considerations we take as key if future work on the PRR, welfare policy and health – but also on populism, nativism, welfare policy and health more broadly – is to avoid some of the pitfalls that have plagued research on populism and the radical right more generally.

Firstly, analysis need to carefully consider the impact of the nativism and authoritarianism of the PRR and not overemphasize its populist character, and carefully consider what role the populist dimension of radical right (or left) politics actually plays in welfare and health policy. Analyses also should avoid attributing nativist and authoritarian tendencies to populism *per se* and avoid lumping together all populist politics as an undifferentiated threat to welfare and health. In practice, this means that studying the impact of ‘populism’ in isolation – as too much commentary on the COVID-19-pandemic has done – is bound to result in analytical problems as well as normative issues.^16^

 Secondly, analysis of the PRR (or the populist left for that matter) need to be embedded in broader reflections on nativist, populist and other threats to healthcare coming from other parts of the political spectrum, whilst also considering the potentially positive effects of certain kinds of populist politics.

 Thirdly, the consequences of populism and nativism can only be gauged properly if we do not take political rhetoric at face value. Populist rhetoric does not necessarily go hand in hand with an actual championing of the interests of the ‘ordinary people’ (as the Trump presidency proves, to give but one example), nor does welfare chauvinist rhetoric or even policy necessarily have positive impacts on ‘the own people’ (as we have argued above). Research on welfare and health policy and its outcomes needs to pay attention to the complex relations between political rhetoric, policy and their (intended and unintended) outcomes. This is not easy to implement, but requires a nuanced and multifaceted understanding of how politics and policy work and interrelate which would likely depend on interdisciplinarity and the combination of diverse methodologies.

## Ethical issues

 Not applicable.

## Competing interests

 Authors declare that they have no competing interests.

## Authors’ contributions

 Both authors contributed equally to the conception, drafting, and review of this commentary.

## Authors’ affiliations

^1^Vrije Universiteit Brussel (VUB), Brussels, Belgium. ^2^School of Health and Social Care, University of Essex, Colchester, UK.
